# A Framework for Automatic Behavior Generation in Multi-Function Swarms

**DOI:** 10.3389/frobt.2020.579403

**Published:** 2020-12-14

**Authors:** Sondre A. Engebraaten, Jonas Moen, Oleg A. Yakimenko, Kyrre Glette

**Affiliations:** ^1^Department of Informatics, University of Oslo, Oslo, Norway; ^2^Norwegian Defence Research Establishment, Oslo, Norway; ^3^Department of Technology Systems, University of Oslo, Kjeller, Norway; ^4^Department of Systems Engineering, Naval Postgraduate School, Monterey, CA, United States

**Keywords:** swarm (methodology), multi-function, MAP-elites, evolution, Quality-Diversity, Physicomimetics, repertoire, geolocation

## Abstract

Multi-function swarms are swarms that solve multiple tasks at once. For example, a quadcopter swarm could be tasked with exploring an area of interest while simultaneously functioning as *ad-hoc* relays. With this type of multi-function comes the challenge of handling potentially conflicting requirements simultaneously. Using the Quality-Diversity algorithm MAP-elites in combination with a suitable controller structure, a framework for automatic behavior generation in multi-function swarms is proposed. The framework is tested on a scenario with three simultaneous tasks: exploration, communication network creation and geolocation of Radio Frequency (RF) emitters. A repertoire is evolved, consisting of a wide range of controllers, or behavior primitives, with different characteristics and trade-offs in the different tasks. This repertoire enables the swarm to online transition between behaviors featuring different trade-offs of applications depending on the situational requirements. Furthermore, the effect of noise on the behavior characteristics in MAP-elites is investigated. A moderate number of re-evaluations is found to increase the robustness while keeping the computational requirements relatively low. A few selected controllers are examined, and the dynamics of transitioning between these controllers are explored. Finally, the study investigates the importance of individual sensor or controller inputs. This is done through ablation, where individual inputs are disabled and their impact on the performance of the swarm controllers is assessed and analyzed.

## 1. Introduction

Typical applications for swarms are tasks that are either too big or complex for single agents to do well. Although it might be possible to imagine a single complex and large agent that is able to solve these tasks, this is often undesirable due to system complexity or cost. Trying to solve several tasks with optimal performance also adds to the complexity (Brambilla et al., 2013; Bayındır, 2016), as each task may place its own requirements or demands on the system. Requirements for being a good long-distance runner are not the same as being a good sprinter. Similarly, in swarms, the requirements for being good at exploring an area are not the same as for maintaining a communication infrastructure. However, an operator that requires capacity and performance in both tasks has limited options. One way of tackling this challenge could be to launch two swarms, giving each swarm a task and operating them independently. This adds complexity to the operation and doubles the system cost. Another option is to develop a concept for a multi-function swarm.

**Figure 1:**
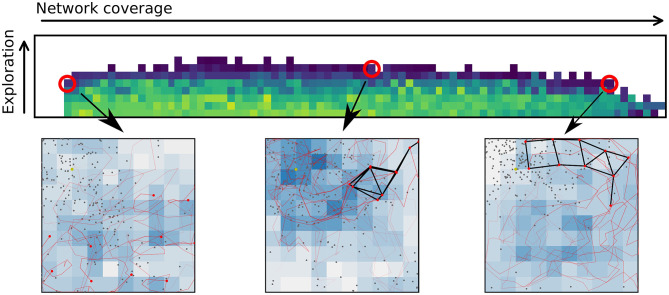
Evolving repertoires of swarm behaviors allows a user to adapt the behavior of the swarm to any desired trade-off between multiple applications or functions by selecting a new behavior from the repertoire. The upper figure shows part of a repertoire where a few selected controllers are highlighted. For each highlighted controller a snapshot of the motion of the given behavior is shown. The snapshots show a birds-eye-view of an area of 100 × 100 m. Red dots indicate agent positions. Black lines indicate connected agents. Gray dots are geolocation estimates, and the intensity of the blue squares indicates how often an area has been visited.

A multi-function swarm, or a swarm that seeks to solve multiple tasks simultaneously, is a novel concept in swarm robotics research. This is different from multitask-assignment (Meng and Gan, 2008; Berman et al., 2009; Jevtic et al., 2011) in that each agent is contributing to all tasks at the same time. It is also different from multi-modal behaviors (Schrum and Miikkulainen, 2012, 2014, 2015), or behaviors to solve tasks that require multiple actions in a sequence (Brutschy et al., 2014). A multi-function swarm tackles multiple tasks at once while retaining some performance on all the tasks simultaneously. Multi-function swarms tackle tasks that are not finite in duration, and as such, require a different approach than typical multi-task assignment. With a relatively low number of agents (compared to e.g., Rubenstein et al., 2012), one cannot afford to have robots assigned to only one or a few of the tasks at a time. Figure 1 shows example swarm behaviors selected from a repertoire with increasing performance in the networking application from left to right. A repertoire provides behaviors with all the possible different trade-offs between applications, resulting in greater flexibility in adapting the swarm behavior to a given scenario.

Most swarm behaviors are defined bottom-up, where the agent-to-agent interactions are specified. For the operator or the user, the desired behavior is commonly on a macroscopic level, or considering the swarm as a whole. Deducing the required low-level rules in order to achieve a specific high-level behavior is a non-trivial problem, and subject to research (Francesca et al., 2014; Jones et al., 2018). Through the use of evolution, this paper seeks to tackle the problem of top-down automated swarm behavior generation.

Previous works show how it is possible to have robots that adapt like animals by using a repertoire of behaviors generated offline (Cully et al., 2015). Similar adaptation techniques should be incorporated into all robotics systems, enabling the recovery from crippling failures or simply a change in the goals of the operator (Engebråten et al., 2018b). A key element of this is that adaptation must happen live, or at least in a semi-live timeframe. Evolving these behaviors online would be the optimal solution, but limited compute and real-time constraints make this infeasible.

Instead of deriving new behaviors on the fly, a swarm could be based on behavior primitives. A behavior primitive is a simple pre-computed behavior that solves some task or sub-task. In this paper, each evolved behavior is considered a behavior primitive. Each behavior represents a full solution to the multi-function behavior optimization problem, however they differ in trade-off between the applications. A swarm agent could easily contain many of these behavior primitives. This would allow the swarm to update its behavior on the fly, as the circumstances or requirements as given by the operator are updated. This negates the need for a full online optimization of behaviors. This is similar to the way a sequence of chained evolved behaviors are used to produce complex sequential behaviors in Duarte et al. (2014). An example would be a search-and-rescue scenario starting with an inactive or idle type behavior primitive. This could be followed by a behavior primitive actively searching for someone missing. Once the missing person or persons were found, the swarm could change to a behavior primitive providing aerial visual coverage and at the same time, a communication network.

A good set of behavior primitives that are easily understandable and solves common tasks goes a long way toward reducing the need for human oversight (Cummings, 2015). A common problem with scaling swarms or multi-agent systems is that the number of humans required to operate the system scales linearly with the number of agents or platforms (Wang et al., 2009; Cummings, 2015). This does not in a good way allow the swarm to be a capability multiplier, as it should be.

Allowing the operator to choose from a set of predefined high-level behavior primitives stored onboard would greatly reduce the need for micromanagement and might break the linear relation between number of agents and operators. By using a Quality-Diversity method (Pugh et al., 2016; Cully and Demiris, 2017) to optimize, it is possible to use the repertoire itself to gleam some insights into the performance for individual behaviors.

In this paper the Quality-Diversity method MAP-elites (Mouret and Clune, 2015) is employed. MAP-elites is used to explore the search space of possible swarming behaviors. The repertoire is a direct way to allow an operator an intuitive view of the potential different trade-offs between multiple applications or functions. The use of a repertoire enables the operator to make informed decisions and highlights costs associated with the choice of behavior in an intuitive manner. In combination with a swarm behavior framework based on physical forces (Engebråten et al., 2018b), this provides a robust and expandable system that has the required level of abstraction to be possible to transfer to real drones.

Three tasks are explored: area surveillance, communication network creation and geolocation of RF emitters. Each task induces new requirements onto the swarm behavior. For instance, covering an area in the surveillance task requires agents to be on the move. Coverage in the communication network application increases if agents are stationary, keeping a fixed distance to each other. It is the combination of these requirements that make this a challenging swarm use-case.

By using a well-known RF geolocation technique based on Power Differential Of Arrival (PDOA) it is possible to give estimates of location for an unknown uncooperative RF emitter (Engebråten, 2015). Previously published works on PDOA (Engebråten et al., 2017) allows this method to be applied also to energy and computationally limited devices. Through extensive simulations the performance of this concurrent multi-functional swarm is evaluated, and the relevance of neighbor interactions examined. Finally, it is shown that the behaviors can indeed be used as behavioral primitives, i.e., building blocks for more complex sequential behavior or as commands from an operator.

The contributions of this paper are an extensible and rigorous framework for multi-function swarms, incorporating automated behavior generation and methods for analyzing the resulting behaviors. This is a major extension of previous works (Engebråten et al., 2018b). Through the use of ablation, or the selective disabling or removal of parts of the controller, importance of individual sensory inputs is determined. The simulator used is updated to better reflect the reality and experiences from previous real-world tests (Engebråten et al., 2018a). Through a combination of extensive simulations, new visualizations and a deep analysis of swarm behaviors, insights can be gained which enables more efficient use of limited computational resources for future evolutionary experiments.

Section 2 presents a view on related works. Section 3 present the methods, framework and simulator used in this study. Section 4 presents the finding and results of the simulations. Section 5 provides thoughts and views on the presented results and section 6 concludes the paper.

## 2. Related Works

### 2.1. Controller Types and Methods

Controllers in the literature for swarms vary greatly. Some propose using neural networks for control (Trianni et al., 2003; Dorigo et al., 2004; Duarte et al., 2016a), handwritten rules (Reynolds, 1987; Krupke et al., 2015), or even a combination or hybrid controller structure (Duarte et al., 2014). Common for all of them is that individual robots, or agents, in some way must receive inputs from the environment or other robots. Based on this information each agent decides on what to do next. This is the basis for a decentralized swarm system and allows the swarm to be robust against single point failures.

The controller structure in this work is an extension upon artificial potential fields (Kuntze and Schill, 1982; Krogh, 1984; Khatib, 1986). Artificial potential fields was originally a method of avoiding collisions for industrial robot control. Additional research allowed this method to be applied to general collision avoidance in robotics (Vadakkepat et al., 2000; Park et al., 2001; Lee and Park, 2003). Generally, potential fields can be said to be a part of the greater superset of methods relying on artificial physics based swarm behaviors. Artificial physics based swarm behaviors is a set of methods that employ artificial physical forces acting between agents, which in turn give rise to the behavior of the swarm as a whole.

A specific type of artificial physics based swarm behaviors is Physicomimetics (Spears et al., 2004; Spears and Spears, 2012). Physicomimetics views each agent as a physical point-mass in a molecular dynamics simulation (Spears et al., 2004). Physical forces, for instance gravity, act between and upon each agent. By varying parameters for the individual forces, the swarm might be made to vary in behavior from a liquid, filling the available space, to a solid, retaining a fixed internal structure. Authors of Physicomimetics also show how the framework can be successfully applied to real-world robots, enabling a swarm of seven robots to reproduce similar results to those found in simulations (Spears et al., 2004).

### 2.2. Evolution of Controllers

Evolution of controllers is a common way of tackling the challenge of automated behavior generation (Francesca et al., 2014; Jones et al., 2018). Evolving a set of sequential behaviors allows agents to tackle multi-modal tasks (Schrum and Miikkulainen, 2012, 2014, 2015). Similarly, evolving behavior trees allow for the evolution of controllers that can easily be understood by human operators (Jones et al., 2018).

Using evolution offline, only time and available computation power limits the complexity of the problems that can be tackled. Online embodied evolution is more limited in the problem complexity, but allows for the behaviors to evolve *in-vivo* or in the operating environment itself (Bredeche et al., 2009; Eiben et al., 2010). Using embodied evolution is a way of allowing robots to learn on the fly, but also to remove the reality gap as agents are tested in the actual environment they operate in. Combining testing of behaviors in a simulator, such as ARGoS (Pinciroli et al., 2011, 2012), with some evolution on real robots to fine-tune behaviors (Koos et al., 2012) improves performance of evolved behaviors while retaining the benefit and speed of offline evolution (Miglino et al., 1995).

Evolving a large repertoire of controllers or behaviors before the robot is deployed might allow it to recover from otherwise crippling physical and hardware faults (Cully et al., 2015; Mouret and Clune, 2015; Cully and Mouret, 2016). Extending on this concept it is also possible to use evolved behaviors to control complex robots as in EvoRBC (Duarte et al., 2016c). When evolving controllers it is important to consider the properties of the evolutionary method chosen. In particular, Quality-Diversity methods perform better with direct encodings than indirect (Tarapore et al., 2016), and might struggle when faced with noisy behavior characteristics or fitness metrics (Justesen et al., 2019). Challenges with noise in traditional evolutionary optimization have been documented well (Cliff et al., 1993; Hancock, 1994; Beyer, 2000; Jin and Branke, 2005). However, as the method MAP-elites is fairly new the effect of noise has not been reviewed to the same extent.

### 2.3. Real-World Applications of Swarms

The ability to operate not only a single Unmanned Aerial Vehicle (UAV) but multiple UAVs is beneficial (Bayraktar et al., 2004). Multiple UAVs may offer increased performance through task allocation (How et al., 2004). A controlled indoor environment allows many swarm concepts to be evaluated without the constraints and uncertainty outdoor tests might bring (Hsieh et al., 2008; Lindsey et al., 2012; Kushleyev et al., 2013; Preiss et al., 2017; Schuler et al., 2019). However, finding a way to move swarms out of the labs and into the real world allows for the verification of early bio-inspired swarm behaviors (Reynolds, 1987), and the effect of reduced communication can be investigated (Hauert et al., 2011). Flocking can also be tested on a larger scale than previously possible (Vásárhelyi et al., 2018).

Outdoors, the potential applications for swarms are many. Swarms could provide a communication network, as is the case in the SMAVNET project (Hauert et al., 2009, 2010). Teams or swarms of UAVs may be used to survey large areas (Basilico and Carpin, 2015; Atten et al., 2016). SWARM-BOT shows how smaller ground-based robots can work together to traverse challenging terrain (Mondada et al., 2004). Pushing boundaries on what is possible, scaling a swarm still presents a challenge, but ARSENL shows that a swarm of 50 UAVs is possible in live flight experiments (Chung et al., 2016). The main challenge, apart from logistics (Mulgaonkar, 2012) in these large experiments, is that of communication and maintaining consensus (Davis et al., 2016). A new frontier for outdoor swarming might be to incorporate heterogeneous platforms with wide sets of different capabilities, further extending the number of applications for the swarm (Dorigo et al., 2013). Swarms of Unmanned Surface Vehicles (USVs) might also prove valuable in environmental monitoring of vast maritime areas (Duarte et al., 2016a,b).

## 3. Methods

### 3.1. A Tri-functional Swarm

The proposed framework uses evolution to automatically create a large set of swarm behaviors. This set of multi-function swarm behaviors is generated based on high-level metrics that measure performance in each application. The core of the framework is the combination of evolutionary methods with a directly encoded controller based on a variant of Physicomimetics, or artificial physics (Spears et al., 2004). This allows the framework to produce a varied set of swarm behaviors. Three applications were chosen to evaluate the framework: area surveillance, communication network creation and geolocation of RF emitters. The first two were introduced in previous works (Engebråten et al., 2018b), while the combination with geolocation of RF emitters is new in this paper.

Making a framework for a multi-function swarm requires development and research into controllers for swarm agents, adaptation of existing evolutionary methods to this task, a suitable simulator to test the proposed swarm behaviors, and realistic assumptions about the capabilities of each individual swarm agent. This section will go into additional details about each of these, starting with the structure of the controllers for each agent.

### 3.2. Simulator Setup

These experiments employ an event-based particle simulator, which processes time based on a series of discrete events instead of fixed time steps. Every agent is modeled as a point mass with limits on maximum velocity and acceleration. A modular architecture allows the simulator to be easily expanded with new sensor, platforms or features. Using the Celery framework for distributed computation allows for task-based parallelization. The full source code for the simulator setup used can be found at GitHub^1^.

Each agent is assumed to be equipped with a radio for communication with other agents and the ground, a camera, and a simple Received Signal Strength (RSS) sensor. All of these are both small in size and weight and constitute a feasible sensor package for a UAV. For these experiments the agents are assumed to have a downward facing camera that can capture the ground below and look for objects of interest. To further simplify the simulation, the simulated environment does not emulate internal/external camera geometry and instead simply assumes that the area of interest can be divided into cells. This is an abstraction only used to simulate a camera; each agent can move freely and continuously across the entire area of interest. Each cell is smaller than the area covered by the camera at any given time, this leads to oversampling of the area. If small enough and a large enough number of cells are used, the method would make it likely that the entire area is covered.

The communication radio is dual-use and acts as both the interlink for the agents (agent-to-agent communication) and the communication channel with the ground control station or other entities on the ground. In previous live-flight experiments WiFi was used (Engebråten et al., 2018a). Newer unpublished experiments have employed a mesh radio which makes it possible to remove the need for a central WiFi router, and as such, furthers the concept of a swarm.

Compared to previous works (Engebråten et al., 2018b), a more conservative vehicle model is employed. Maximum acceleration was reduced to 1.0 m/s^2^ and max velocity was set to 10.0 m/s. This was based on the results of previous real-flight experiments (Engebråten et al., 2018a) and is a way to compensate for the slower reaction time of the physical vehicles.

Furthermore, it was found that the range of the controller parameters determining the behavior of the platform were in many cases too high to be readily employed on real UAVs. This led to oscillating behaviors where the time delay in the physical system could not keep up with the controller.

### 3.3. Applications in the Multi-Function Swarm

**Figure 2:**
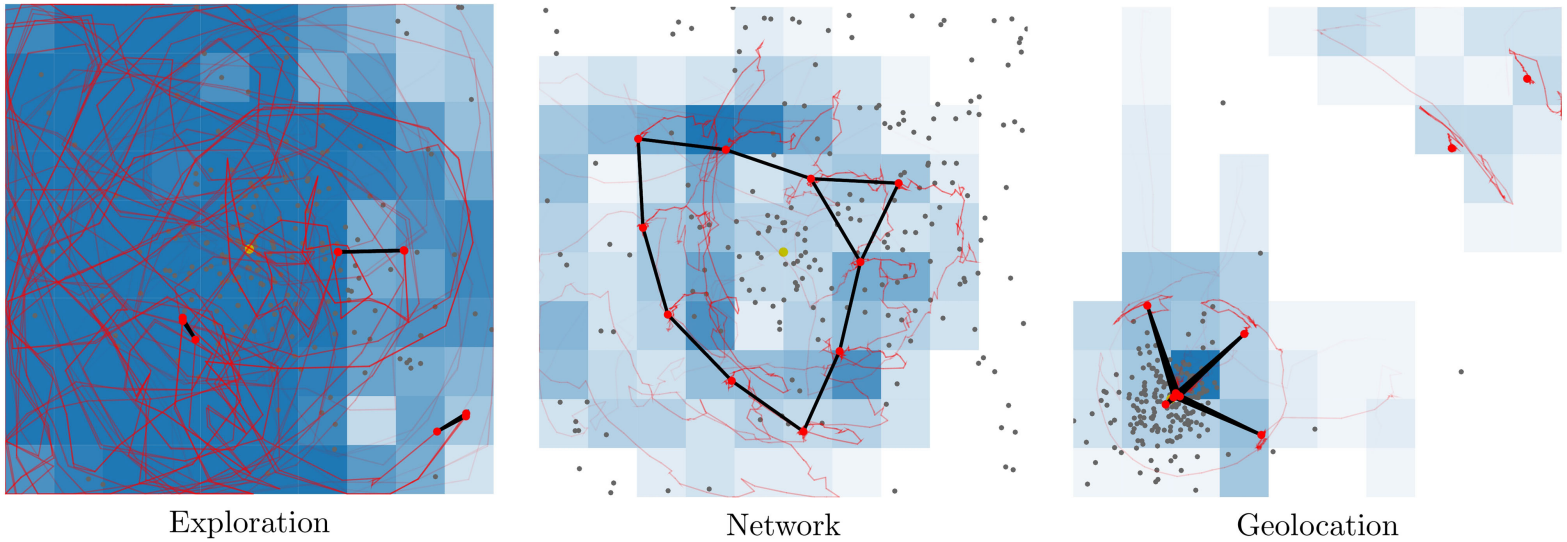
The multi-function swarm is optimized on three applications; exploration, network creation, and geolocation (from left to right). Each application requires distinct behaviors for optimal performance. Red dots indicate each swarm agent.

A swarm of UAVs might in the future be used to provide real-time visual observations over large areas. On a conceptually high level these can be considered a potential replacement for a fixed security system, providing both better coverage, a more flexible and adaptable setup and the ability to react to new situations with ease. The downside is that today they require more maintenance and logistics, as well as more operator oversight. In this work a simplified area surveillance scenario is used as one of the applications. Each agent is equipped with a camera and tasked to explore an area. Exploration is measured by dividing the area of interest into a number of cells. The agents in the swarm seek to explore, or cover, all the cells as frequently as possible (see left part of Figure 2). The median visitation count across all cells in the area is used to measure performance in the area surveillance task. The normalized median is denoted *b*_*e*_. For more information refer to previous works (Engebråten et al., 2018b).

In the absence of a common wireless infrastructure, it is also natural to imagine that the swarm must provide its own communication network in order to relay information back to an operator or a ground control station. This requires the UAVs to continuously be in range for forwarding of data to be possible. Communication between entities far apart may require multi-hops, this is typically solved by routing algorithms which discover, update and maintain new routes as they become available. A simplified scenario for maintaining a communication infrastructure is used as a second application for the swarm (middle part of Figure 2). Swarm behaviors are measured on the ability to maintain coverage and connectivity over a large area. The performance in the communication network task is measured by calculating the area covered by the largest connected subgroup of the swarm, given a fixed communication radius. The normalized area used as the metric is denoted *b*_*n*_. This metric was also used in previous works (Engebråten et al., 2018b) and promotes agents spreading out while maintaining connectivity.

PDOA geolocation is introduced as a third application for the multi-function swarm (right part of Figure 2). Geolocation refers to trying to find the geographic position, or coordinates, of an RF emitter based on sensor measurements. PDOA uses the RSS, or the received power, at multiple different points in space in order to give an estimate of the location of the emitter (right part of Figure 2). PDOA geolocation is a form of trilateration, or more specifically, multilateration in that the sensor readings give an indication of distance (as opposed to direction used in triangulation). A prediction of the emitter location can be made by minimizing *Q*(*x, y*). *Q*(*x, y*) (Equation 1) is an error function that indicates the error compared to Free Space Path Loss model, given that the emitter is at coordinates (*x, y*). In essence, *Q*(*x, y*) compares the differences in RSS at multiple different positions, which is comparable to looking at the difference in distances. *P*_*k*_ and *P*_*l*_ represents the RSS at positions (*x*_*k*_, *y*_*k*_) and (*x*_*l*_, *y*_*l*_), respectively. α is the path loss factor, for these experiments a value of 2.0 is used. Previous works presented a method of providing an estimate of the location of a transmitter using significantly less resources than commonly employed estimators (Engebråten et al., 2017). In this work, *Q*(*x, y*) is sampled at 60 random locations and the location with the least error is used as an estimate for emitter location. This forgoes the local search used in (Engebråten et al., 2017).

(1) $$\begin{array}{l} Q\left(x,y\right)=\sum_{k=1}^{n}\sum_{l=k}^{n}[\left(P_{k}-P_{l}\right)\\ \;-5\alpha \;\log\frac{{\left(x-x_{l}\right)}^{2}+{\left(y-y_{l}\right)}^{2}}{{\left(x-x_{k}\right)}^{2}+{\left(y-y_{k}\right)}^{2}}{]}^{2} \end{array}$$

Over time, multiple estimates of the emitter location are produced by the swarm. The variance of all these predictions are calculated and used as a metric for performance in the geolocation task. The normalized variance is denoted *b*_*l*_. It is important to note that the use of PDOA geolocation has specific requirements on sensor placement to avoid ambiguities that lead to great variance and inaccuracies in the predicted emitter locations. For more information about this, refer to previous works (Engebråten, 2015). In most cases, variance naturally converges toward zero as the mean converges on the true mean.

### 3.4. Controller Framework

Controllers for each swarm agent are based on a variant of Physicomimetics, or artificial physics (Spears et al., 2004). Artificial forces act between the agents and, ultimately, define the behavior of the swarm. Unlike traditional physics there is no limit on the type of forces that can act between agents.

**Figure 3:**
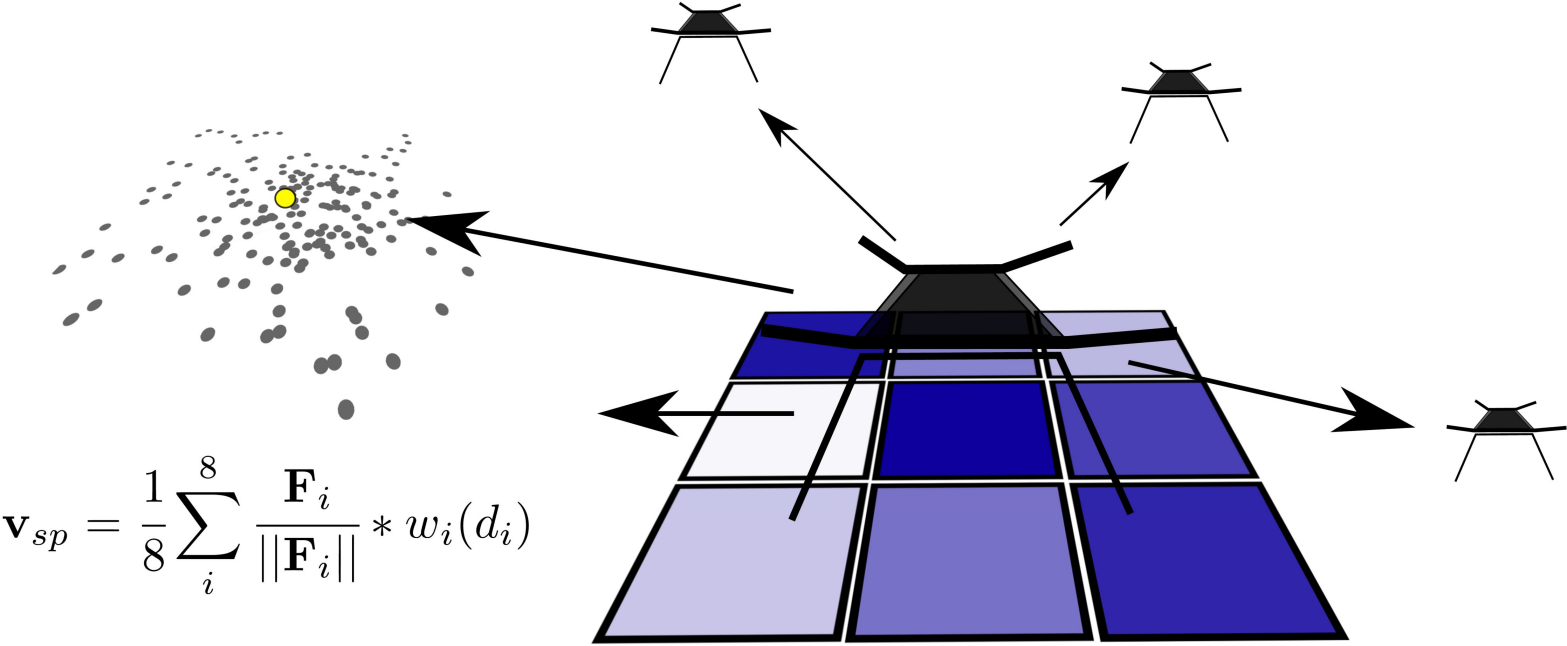
Each agent uses the distance to the six nearest neighbors, the direction to the least visited surrounding square, and the direction and distance to the average of the predicted emitter location. Together, using Sigmoid-Well *w*_*i*_(*d*_*i*_), they form a velocity setpoint **V**_*sp*_.

This type of swarm behavior can easily model most types of repulsive, attractive and relative-distance-holding type behaviors, all depending on the inputs that are given to the controller. The controller is based on distances and directions to (dynamic) points of interest surrounding the agent. For the controller used in this work (Figure 3) eight inputs are used:

(*F*_1_) Nearest neighbor
(*F*_2_) Second nearest neighbor
(*F*_3_) Third nearest neighbor
(*F*_4_) Fourth nearest neighbor
(*F*_5_) Fifth nearest neighbor
(*F*_6_) Sixth nearest neighbor
(*F*_7_) Least frequently visited neighboring square
(*F*_8_) Average predicted emitter location.

**Figure 4:**
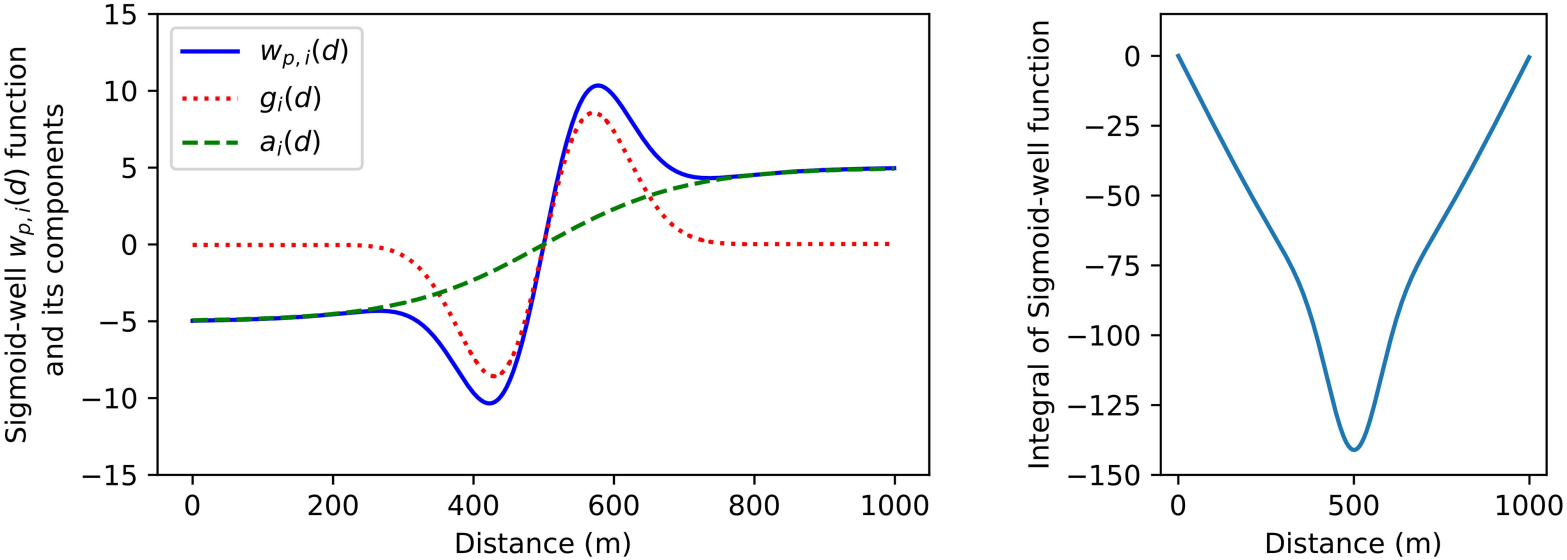
The Sigmoid-Well function used in these experiments. Weight and scale parameters are coupled to the center parameters and spread parameters. This is because the sign of the Sigmoid-Well function changes at the center distance. Each agent minimizes the combined potential of all the contributing forces, indirectly moving to the minimum of the integral function on the right. *k*_*i*_ is 5.0, *t*_*i*_ is −0.1, *c*_*i*_ is 500.0, and σ_*i*_ is 100.

In this work, the force that acts between agents is defined by the Sigmoid-Well function. This function is comprised of two parts *a*_*i*_(*d*_*i*_) (Equation 3) and *g*_*i*_(*d*_*i*_) (Equation 2). *a*_*i*_(*d*_*i*_) is a distance dependent attraction-replusion force. *g*_*i*_(*d*_*i*_) is the gravity well component, which can contribute with distance holding type behaviors. These functions are defined by four parameters: the *k*_*i*_ weight, the scale parameter *t*_*i*_ the *c*_*i*_ center-distance and the σ_*i*_ range parameter. The *k*_*i*_ weight determines the strength of the attraction-repulsion force. The scale parameter *t*_*i*_ defines the affinity toward the distance given by the center-distance *c*_*i*_. The range parameter σ_*i*_ can increase or decrease the effective range of the gravity well, by lengthening the transition around the center distance *c*_*i*_. Together *a*_*i*_(*d*_*i*_) and *g*_*i*_(*d*_*i*_) form the Sigmoid-well function *w*_*i*_(*d*_*i*_) (Equation 4). An example of the shape of each of these components can be seen in Figure 4, which shows how the function *w*_*i*_(*d*_*i*_) can be set to enact a repulsion/attraction force, in addition to a preference for holding a distance of 500 m.

(2) $$\begin{array}{rcl} g_{i}\left(d_{i}\right) & = & -2t_{i}\left(d_{i}-c_{i}\right)e^{-{\left(d_{i}-c_{i}\right)}^{2}/\sigma_{i}^{2}} \end{array}$$

(3) $$\begin{array}{rcl} a_{i}\left(d_{i}\right) & = & k_{i}\left(\frac{2}{1+e^{-\left(d_{i}-c_{i}\right)/\sigma_{i}}}-1\right) \end{array}$$

(4) $$\begin{array}{rcl} w_{i}\left(d_{i}\right) & = & a_{i}\left(d_{i}\right)+g_{i}\left(d_{i}\right) \end{array}$$

(5) $$\begin{array}{rcl} {\text{v}}_{sp} & = & \frac{1}{8}\overset{8}{\underset{i}{\sum }}\frac{{\text{F}}_{i}}{\left||{\text{F}}_{i}|\right|}w_{i}\left(\left||{\text{F}}_{i}|\right|\right) \end{array}$$

The eight inputs are combined by scaling them with the Sigmoid-Well function *w*_*i*_(*d*_*i*_) before summing the result to form a single velocity setpoint **V**_*sp*_ (Equation 5). **F**_*i*_ is the distance delta vector from agent position to sensed object position. **V**_*sp*_ is calculated based on **F**_*i*_ and *w*_*i*_(*d*_*i*_).

For each input there are four parameters: a weight *k*_*i*_, a scale *t*_*i*_, a center *c*_*i*_ and a range σ_*i*_. With a total of eight inputs this gives 32 parameters. The least visited neighboring square input is slightly different from the rest of the sensory inputs. This input gives only direction information to the controller and not a distance. The controller handles this by only weighting this input and not applying the distance dependent Sigmoid-Well function. This means that in practice, for each controller, only 29 parameters make an impact on the swarm behavior. Assuming a fixed 4 bytes per float this means that a single megabyte could hold over 8,000 different swarm behaviors. This makes it possible for all but the most limited types of swarm platforms to retain many of these behaviors.

Compared to previous works (Engebråten et al., 2018b), the ranges of the weight and scale parameters were reduced. For these experiments, the weight parameter is limited from −5.0 to 5.0 and the scaling parameter is limited from −0.5 to 0.5. It should be noted that due to the form of the Sigmoid-Well function (Engebråten et al., 2018b) the scaling parameter is stronger and has a smaller allowable range.

### 3.5. Evolving Controllers

The Quality-Diversity method MAP-elites is used to evolve swarm controllers. MAP-elites seeks to explore the search space of all possible controllers by filling a number of characteristics bins spanning the search space of all controllers (Mouret and Clune, 2015). Variation of solutions in MAP-elites is done by mutations. The MAP-elites algorithm used in this work is described in pseudocode in Algorithm 1. In this work, mutation is performed by first selecting a parameter at random then adding a Gaussian perturbation with a mean of 0.0 and a variance that is 10% of the range of the parameter chosen (Table 1). Only a single parameter is changed per mutation. The behavior characteristics tuple *b*′ consists of (*b*_*e*_, *b*_*n*_, *b*_*l*_) and is used as an index to the repertoire P. Adaptation to fill new bins might require multiple sequential mutations in order to move from one characteristics bin to another. This type of adaptation might be challenging for the MAP-elites algorithm, as it also requires that the solution outperforms existing solutions along the path required to reach the new unoccupied bins.

**Algorithm 1:** MAP-Elites Algorithm with batch evaluation.

1:	**procedure** EvolveRepertoire	
2:	P ← *empty repertoire*	
3:	**for** *i* = 1 to *n* **do**	⊳ for *n* generations
4:	**if** i == 1 **then**	
5:	***X*′** ← *randomly selected set of solutions*	
6:	**else**	
7:	***X*** ← selected set of random solutions from P	
8:	***X***′ ← permute(***X***)	⊳ mutate a single parameter in each solution in ***X***
9:	(***B***′, ***F***′) ← evaluate(***X***′)	⊳ evaluate batch ***X*′** and calculate characteristics and fitness
10:	**for** *(x*′,*b*′,*f*′*)* in (***X***′,***B***′,***F***′) **do**	⊳ for each tuple of solution, characteristic and fitness
11:	**if** $P\left(b^{\prime }\right)==\emptyset$ or $P\left(b^{\prime }\right)<f^{\prime }$ **then**	⊳ if bin at *b*′ is empty or existing solution is worse
12:	$P\left(b^{\prime }\right)\leftarrow x^{\prime }$	⊳ place solution *x*′ in P according to characteristics *b*′
13:	**return** P	⊳ return final repertoire P

**Table 1:** Range and mutation for each type of controller parameter.

	**Range**	**Permutation step**
Weights (*k*_*i*_)	[−5, 5]	N (0.0, 1.0)
Scales (*t*_*i*_)	[−0.5, 0.5]	N (0.0, 0.1)
Centers (*c*_*i*_)	[0.0, 1000.0]	N (0.0, 100.0)
Ranges (σ_*i*_)	[0.0, 100.0]	N (0.0, 10.)

As part of the evolutionary process, three behavior characteristics and a fitness metric are used. As mentioned in subsection 3.3 the characteristics or metrics are: the median visitation count across all the cells in the area of operation, the area covered by the communication radii of the largest connected subgroup of the swarm, and the variance in predicted locations for the single RF emitter being sought. For the networking application each agent is assumed to have a fixed communication radius of 200 m. All characteristics are normalized to the range [0.0,1.0]. Fitness *f* is calculated in a deterministic manner based on the scales parameter vector (**t**) and weights parameters vector (**k**) of the controller (Equation 6). These parameters determine the magnitude of the output of the controller and as such correlate well with the motion that can be expected from the controller. In order to avoid sudden (high) acceleration and reduce battery consumption, behaviors are optimized to minimize motion and maximize *f*.

(6) $$\begin{array}{rcl} f & = & \frac{1}{\left||\text{t}||+||\text{k}|\right|} \end{array}$$

## 4. Experiments and Results

A series of experiments are conducted to explore the evolutionary process itself, the viability of using the evolved swarm behaviors as behavior primitives, the effect of noise on an evolutionary process using MAP-elites and finally, the value of disabling certain controllers inputs are examined. The evolution of a single repertoire takes around 16 hours on a cluster running 132 threads; thus ~2,112 CPU hours per repertoire. Total simulation time for the ablation study (subsection 4.4) is ~152,064 CPU hours or 17.6 CPU years.

### 4.1. Repertoire Evolution

All the repertoires in this work have 10 exploration bins × 100 network bins × 10 localization characteristics bins. Previous works examined the two applications of exploration and network creation using a repertoire of 10 exploration bins × 100 network bins (Engebråten et al., 2018b). In this work, repertoires are extended with another dimension featuring 10 localization characteristics bins. The new dimension has only 10 bins to keep the repertoire size manageable. Discretization and the definition of the bins in MAP-elites plays a major role in the algorithm's ability to successfully find a good and non-sparse repertoire. For instance, the exploration metric, which is the median visitation count across all cells in the area, is carefully designed in order to avoid discretization issues which cause certain bins to never be filled due to the fact that the median is always a whole number.

**Figure 5:**
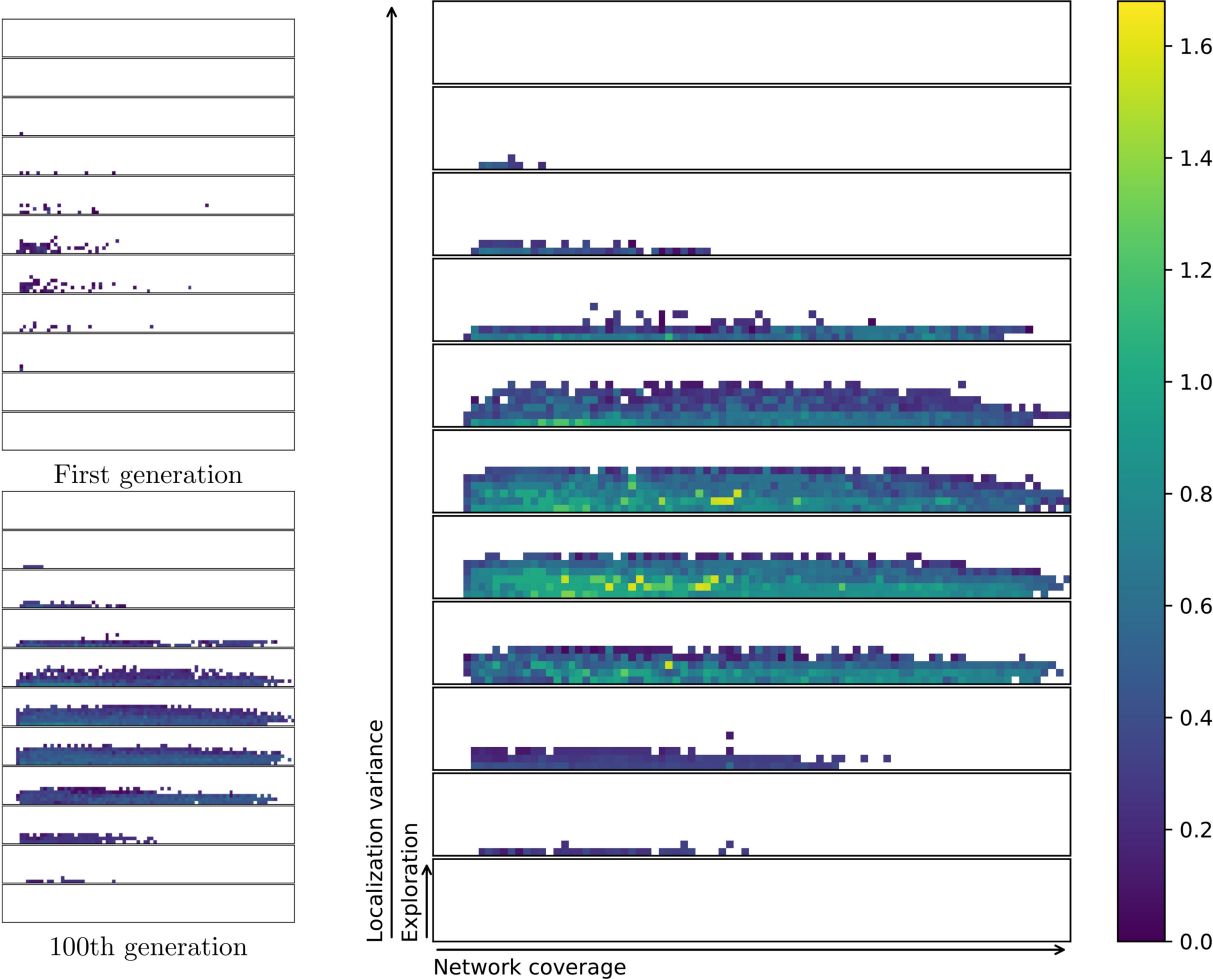
Progression of the evolution of one repertoire. Repertoires are visualized by slicing the three-dimensional behavior space along all three axes (*b*_*e*_, *b*_*n*_, *b*_*l*_), this allows higher dimensions to be flattened for easier visualization. Exploration, network coverage and localization variance are normalized to the range of [0.0,1.0]. Behavior space is divided into 10 exploration bins × 100 network bins × 10 localization characteristics bins. Bottom slice covers localization variance from [0.0,1.0). A total of 11 slices are shown in the image; the top slice [1.0,1.1) is overflow in case any behavior should achieve a localization variance of 1.0 or above. This is technically possible, but highly unlikely due to floor-rounding and all characteristics being normalized. The right repertoire is the final result after 200 generations. Brighter yellow indicates better solutions, as measured by fitness.

The characteristics bins are filled in during 200 pseudo-generations, each evaluating and testing 200 individuals, resulting in the final repertoire. Figure 5 shows the progression of evolving a single repertoire. A total of eight independent evolutionary runs are conducted. On average, across 8 runs, the evolution resulted in 2,031 solutions in each repertoire. This represents a coverage of 20.3% with a standard deviation in number of solutions of 101.1. As can be seen in Figure 5, the first half of the evolution fills out most of the repertoire. Solutions are further optimized, and the repertoire is slightly extended during the second half of the evolutionary process. White or empty spaces may be solutions that are hard to find for the MAP-elites algorithm or simply impossible to attain with the given controller framework. For example, controllers in the top right corner of one of the slices would have very high exploration and very high network coverage. This is likely not feasible as extreme exploratory behaviors would not be able to stay within networking distance, given the number of agents in the experiment.

### 4.2. Swarm Behaviors as Behavior Primitives

**Figure 6:**
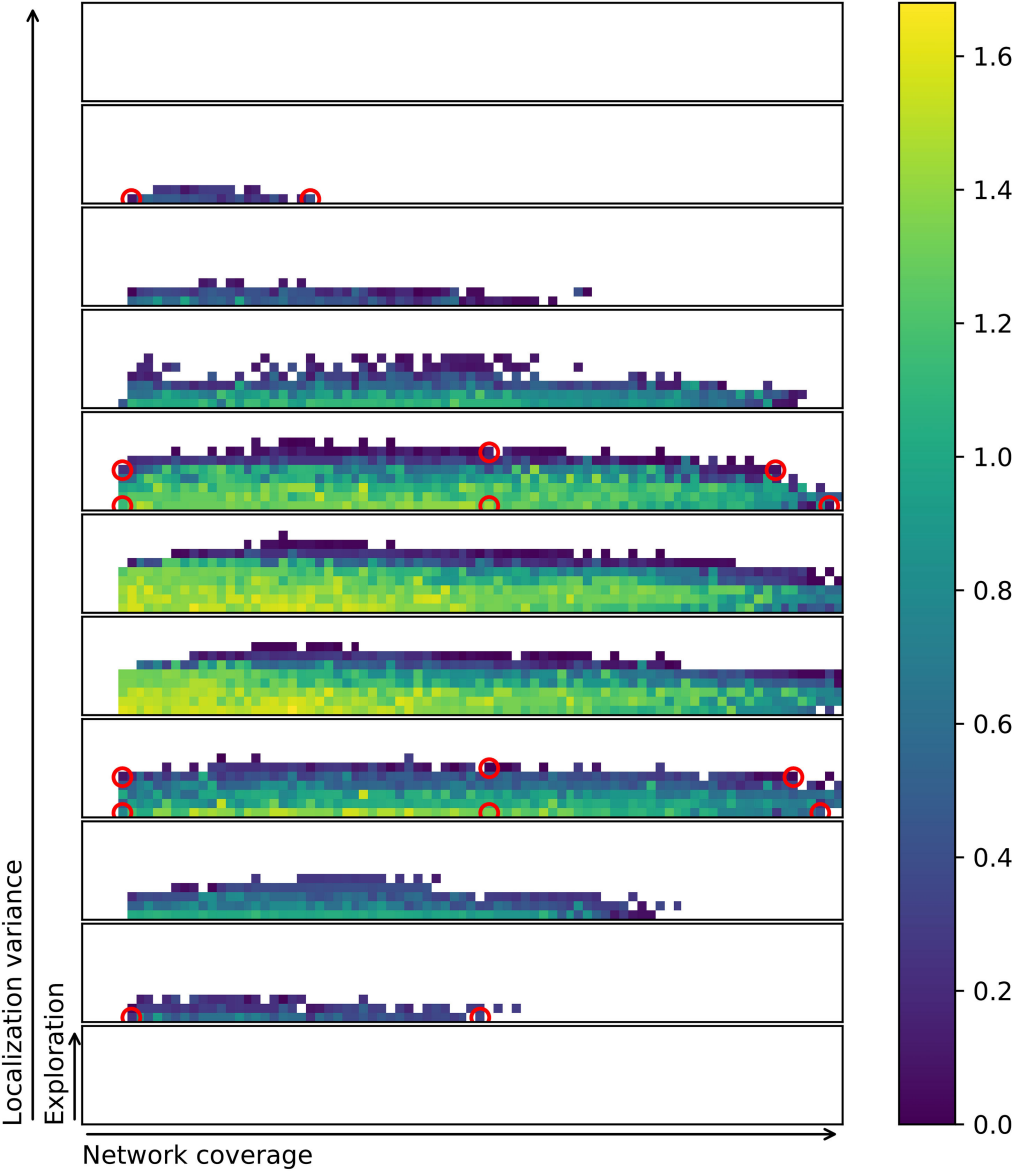
Combined repertoire from eight separate evolutionary runs. Each filled square represents a characteristics bin. Circled controllers are selected for a more in-depth examination. Brighter yellow indicates better solutions, as measured by fitness. For more on characteristics bins and discretization of slices refer to Figure 5.

**Figure 7:**
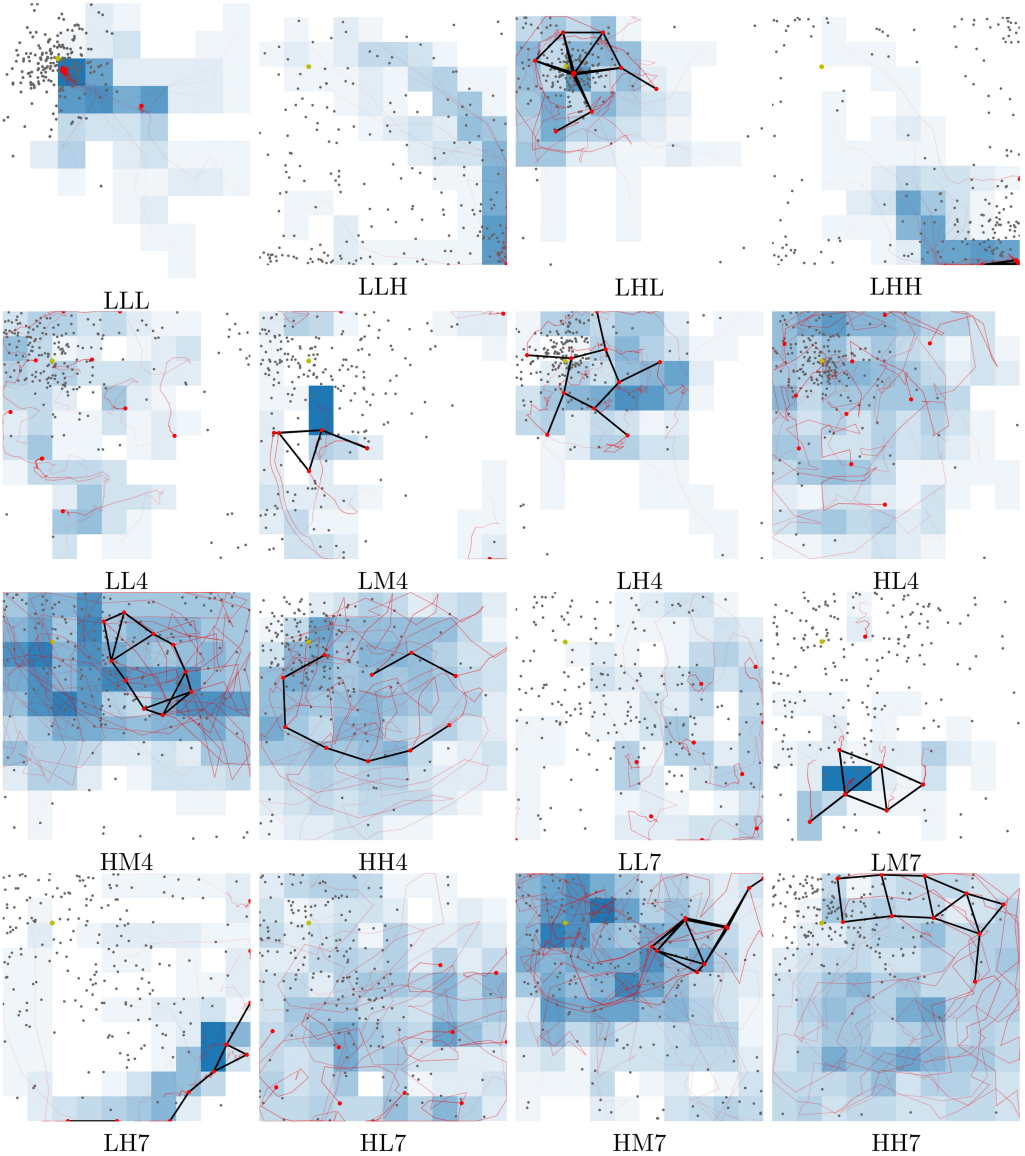
Traces of motion for each behavior highlighted in Figure 6. Each behavior is tested in an area of 100 × 100 m and this figure shows the traces as seen from a birds-eye-view. Labels below each subfigure refer to the behavior locations in the repertoire. **LHL** for instance is a behavior with **L**ow exploration, **H**igh network coverage and **L**ow variance in geolocation predictions. If the last character is a number, this refers to the slice in the repertoire starting at 1 from the bottom. **HL4** for instance is a behavior with **H**igh exploration, **L**ow network coverage and localization variance within slice **4** [0.3,0.4). Red lines indicate the path of the UAVs and black lines are connected UAVs, as determined by communication radius. Gray dots indicate location predictions. Deeper blue squares are more frequently explored. Videos of all the behaviors can be found at https://www.youtube.com/playlist?list=PL18bqX3rX5tT7p94T2_j2B3C4HkiMVZvY.

In this subsection, a subset of the evolved controllers is examined and their viability as behavior primitives are investigated. The best controllers found across all runs are stored in a repertoire (Figure 6). From this repertoire, 16 controllers are selected by visual inspection and examined in greater detail. Selecting behaviors by visual inspection is possible because any repertoire can be flattened by slicing it (Cully et al., 2015). Controllers are selected on the boundary of the feasible controller region, as this is assumed to provide the most extreme set of varied behaviors. Figure 6 shows the location of each of the solutions and Figure 7 shows trace plots of the behaviors.

**Figure 8:**
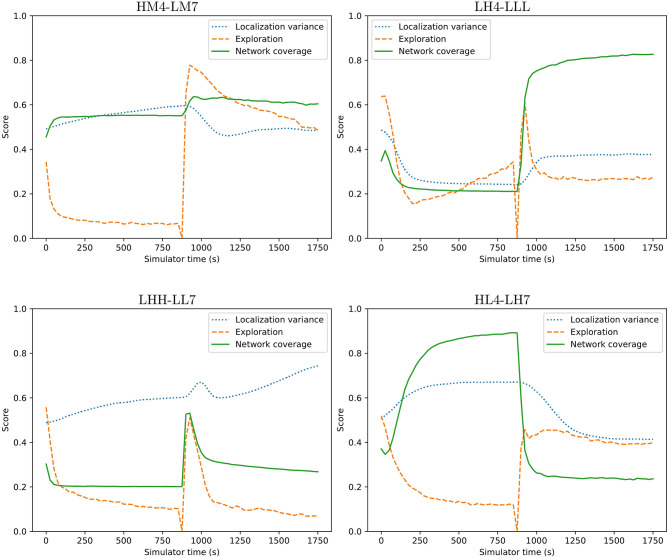
Four example transitions between two behaviors. Transitions happen at time 900. Graphs show averages over 1,000 tests. From Figure 7, four of the possible 240 transitions were selected and shown. For instance, **HM4-LM7** transitions between behavior **HM4** to **LM7**. There is a temporary reduction in exploration score during the transition due to the way exploration is measured and the agents re-organizing to the new behavior.

Transitioning between different behaviors shows whether the behaviors may be used as behavior primitives. The transitions between the selected controllers are examined using a surrogate metric for the exploration behavior characteristic. The exploration metric used when evolving behaviors calculates the median visitation count across all cells in the simulation area. This does not work on a short timescale as the area is too large and the median visitation count rarely gets above 0. Instead, the value of this metric is approximated using the derivative of the total visitation count calculated over the time interval since the metric was last evaluated. This gives a rough indication of how good the behaviors are in the different applications, but is subject to noise. To alleviate the noise in this measurement each transition is tested 1,000 times. The average time series are shown in Figure 8. The figure shows four examples of transitions between behaviors. As far as the controller is concerned, there is no discernible difference between starting from where another behavior left off vs. from a clean simulation as each controller is stateless. This is essential for these behaviors to be applicable as a set of behavior primitives and is what could enable the operator to change the behavior of the swarm on the fly.

From Figure 8 it is possible to see that behaviors can change on the fly, allowing the adaptation to user preferences or requirements. Some behaviors take a couple of minutes in simulation time to stabilize into a steady state. This can be seen from the initial gradual change from time 0. Once the behavior has been allowed to stabilize all metrics eventually converge to a single value before the abrupt change of behavior at time 900. During the change of behavior there is a brief discontinuous phase where the swarm re-organizes into the new behavior. The spike often seen in the exploration metric is due to the surrogate metric and the fact that agents move rapidly in order to adapt their individual behavior to the new requirements. Network coverage and localization is not subject to the same spike in most cases. In the **LHH** to **LL7** transition there is a spike in the network coverage metric, which is likely due to a brief temporary clustering of agents as each agent assumes its new position within the swarm. The swarm is a highly dynamic system and reactive, even though the smooth transitions do not give this impression. The averaging over 1,000 transitions highlights the change while removing the variation between individual runs. Figure 7 has links to videos which better show individual behaviors in detail.

### 4.3. Investigating the Effect of Noise in MAP-Elites

MAP-elites is a greedy algorithm. Every time a solution is mutated it is kept as a part of the repertoire if it fills a void where there previously was no solution, or it is better than the existing solution. It is an excellent property to maintain diversity and allows for better exploration of the search landscape, but also poses a challenge. Many common metrics or fitness functions used in evolutionary optimization are stochastic (Beyer, 2000). This applies to both fitness metrics and behavior characteristics. If a stochastic variable has high variance, but a low mean it still might outcompete a stochastic variable with high mean and low variance. In the case of these experiments, a behavior might get a lucky draw from the metrics used to evaluate performance, resulting in an inferior solution being chosen over a superior solution. This is a challenge, as in many cases, the lesser variability and higher mean may be preferable to the lower mean and greater variability.

**Figure 9:**
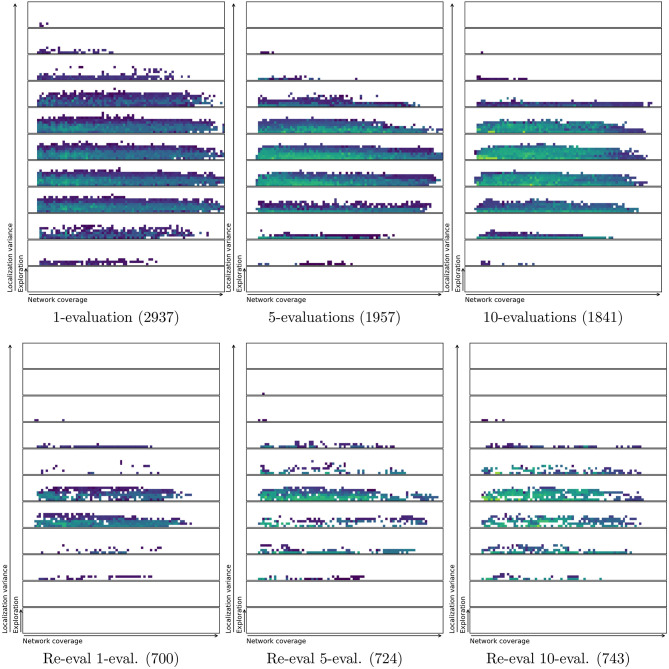
Original (top) and re-evaluated repertoires (bot) for runs using an average of 1, 5, and 10 evaluations per controller in the initial repertoire evolution. Re-evaluation with 100 evaluations per candidate solution results in a large reduction in repertoire size. This indicates that many of the behaviors found by MAP-elites have the same characteristics values when the variance is reduced sufficiently to uncover the true characteristics values. In other words, a large number of the solutions found originally may have been duplicates caused by greedy exploration. The number of solutions in each repertoire is shown in parentheses. Colorbars are the same as for Figure 6, but were removed to minimize clutter.

In order to test if a repertoire is reproduceable an entire repertoire is re-evaluated using more evaluations per solution or behavior. This gives a clear visual indication of whether the solution stays in the same characteristics bin or moves around. Figure 9 and Table 2 shows that there is a noticeable reduction in the number of solutions in the repertoires as they are re-evaluated. The figure shows an overview of repertoires evolved and re-evaluated with a single, 5 evaluations and 10 evaluations per controller. It is important to note that using more evaluations in the initial evolution results in a smaller repertoire. This is likely due to reduced variance in the characteristics metrics as a result of averaging across multiple evaluations (Justesen et al., 2019). However, the smaller resulting repertoire is probably closer to the true shape of the space of all feasible swarming behaviors. Using only a single evaluation results in a repertoire with 2,937 solutions, while 5-evaluations results in only 1,957 solutions. The further reduction with 10-evaluations is much smaller, with the final repertoire having 1,841 solutions.

**Table 2:** Overview of results of re-evaluation of controller repertoires.

	**1-eval repertoire**	**5-eval repertoire**	**10-eval repertoire**
Original	2,937	1,957	1,841
Re-eval 20-eval	823 (28.0%)	857 (43.8%)	859 (46.7%)
Re-eval 50-eval	744 (25.3%)	759 (38.8%)	800 (43.4%)
Re-eval 100-eval	700 (23.8%)	724 (37.0%)	743 (40.4%)

It is important to note that re-evaluating using 20 evaluations per solution cannot be compared to evolving a repertoire with 20 evaluations per solution. During the evolutionary process, a total of 40,000 solutions are tested. During re-evaluation, only the 2,000–3,000 solutions in the repertoire are re-evaluated. As such, it is natural that the re-evaluated repertoire contains a lot fewer solutions as it was not given time to search for solutions to fill all the characteristics bins.

Increasing the number of evaluations seems to have an effect by producing a repertoire that is more correct, or closer to the true underlying shape. However, the effect is also diminishing: going from 5 to 10 evaluations has little effect. Therefore, all other experiments in this work used 5-evaluations per individual.

Table 2 shows that the number of evaluations used in the re-evaluation step is not that important. The greatest reduction in repertoire size is found when using the highest number of evaluations in the re-evaluation step (100 evaluations per solution). However, the effect of increasing the number of evaluations on the robustness in the initial repertoires can also be clearly seen when re-evaluating the repertoire with only 20 evaluations per individual.

**Figure 10:**
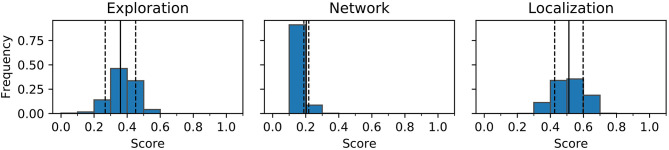
Example of a probability distribution of characteristics over 1,000 simulations of the same controller. Counts are normalized to sum to one across all 10 bins for each characteristic. The solid line is the mean of samples, dotted lines indicate one standard deviation.

To further investigate the challenge of reproducing behaviors and repertoires a single behavior (HL7) is re-evaluated 1,000 times and the probability distribution of the behavior characteristics are shown in Figure 10. In the experiments conducted in this paper the fitness can be computed from the genome deterministically, so only the three behavior characteristics (exploration, network coverage, and localization variance) are reviewed.

Figure 10 shows that the distributions for each of the three metrics resembles a normal distribution in most cases. Variation, as indicated in the figure, can cause a behavior to seemingly move between characteristics bins when re-evaluated, this is believed to the primary cause of the reduction in size of the repertoire.

**Figure 11:**
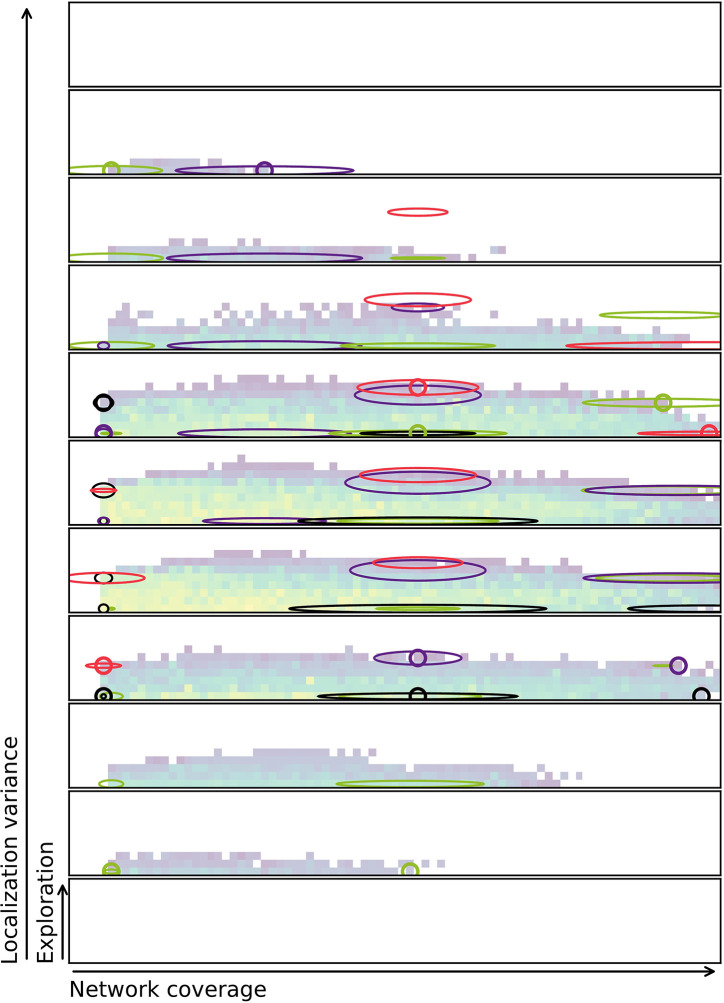
Repertoire with diagonal covariance ellipses plotted for selected behaviors. The small circles indiciate the location of the behavior in the final repertoire. Slices of the ellipse are shown in the same color as the circle indicating the behavior. Note that behaviors at the edge of the repertoire also often are at the edge of the ellipse. This indicates that behaviors with these characteristics may be hard to come by. For more on characteristics bins and discretization of slices refer to Figure 5. Colorbar is the same as for Figure 6, but was removed to minimize clutter.

A challenge that remains is to successfully quantify the properties of the solutions. If the measure has stochastic properties, the exact same solution might fit in multiple bins in the repertoire. This again means that there is uncertainty whether the evolutionary method captures the true shape of the behavior space or not. To visualize this, Figure 11 shows a combined repertoire over eight evolutionary runs with estimated diagonal covariance ellipses plotted as slices in 3D. As can be seen from the figure, the behavior is not always found at the center, or mean, of the distribution. The diagonal covariance ellipses (indicating one std. dev.) are estimates generated by evaluating each of the selected controllers 1,000 times and calculating variance and mean over these runs.

Figure 11 shows how solutions might jump between characteristics bins if re-evaluated. It also indicates that many of the solutions that are in the repertoire are at the very edge of the potential range of values that the characteristics may take on. This suggests the idea that MAP-elites might be biased toward accepting solutions that have a high variance, as they sometimes get lucky and provide a solution for a hard to reach characteristics bin.

### 4.4. Ablation Study of the Effect of Controller Inputs

The proposed controller has eight inputs that determine the action of a swarm agent at any given time. The inputs are distance and direction to the nearest six neighbors, least frequently visited square and average predicted emitter location. In previous works (Engebråten et al., 2018b), a simpler parametric controller with only four inputs was employed, as well as a controller with only scalar weights, which did not enable the agents to evolve holding distance type behaviors. In this work, the distance and direction to another three neighbors was added. This was done in the interest of improving the performance of the controller in the three given applications. To quantify the effect of this change and the ability of the evolutionary process to find good swarm behaviors, an ablation study is performed. Individual inputs are disabled, which allows the effect of each input to be examined separately. Ablation refers to the selective disabling or removal of certain parts or a larger object in order to investigate the effect this might have.

**Figure 12:**
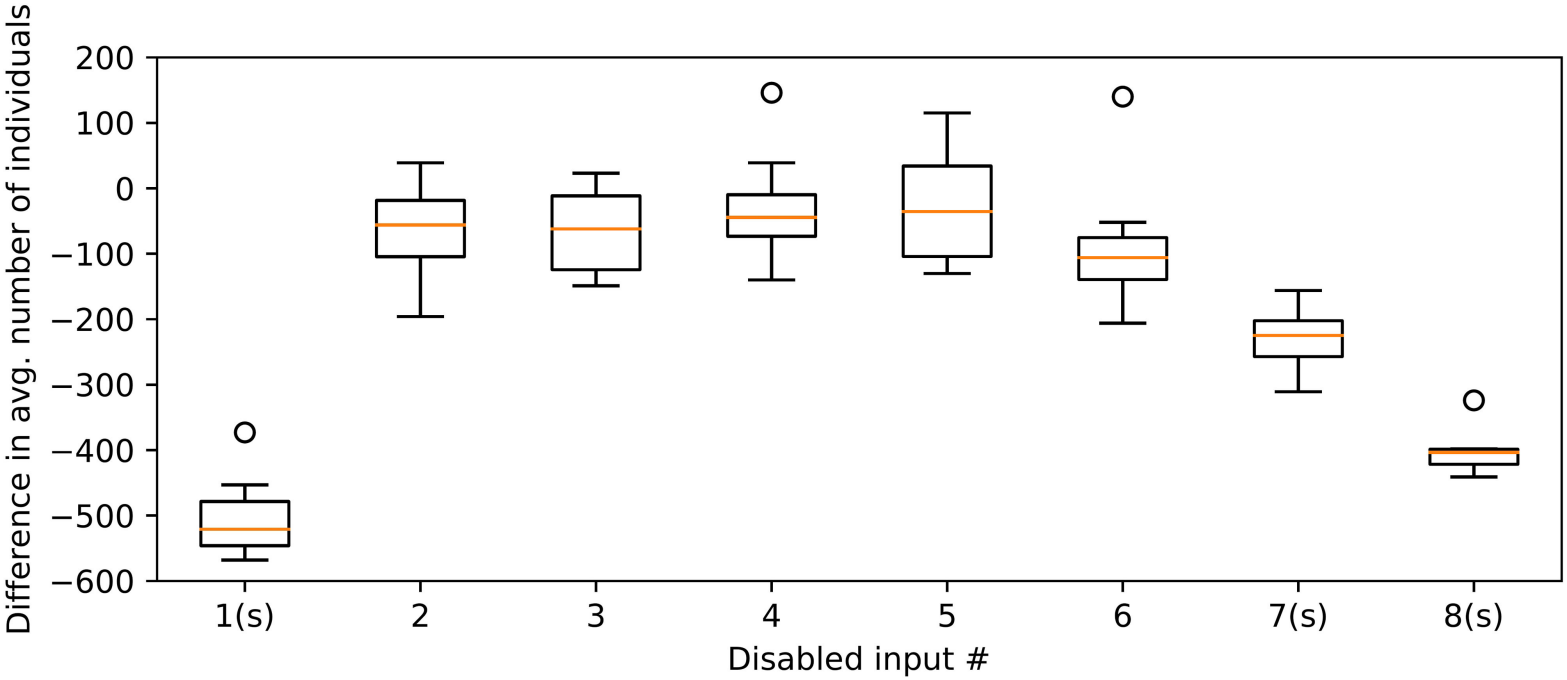
Difference in individual count when disabling one input at the time to the swarm controller. An (s) after the X-axis label indicates statistical significance (*P* < 0.01) in Rank-Sum test, comparing against all inputs enabled. Tests remain significant when a Bonferroni-correction is applied. This is done to compensate for any potential multiple comparison errors. Circles indicate outliers outside the range of the whiskers. This box plot uses whiskers that may extend up 1.5 times of the interquartile range; any data points outside this range are considered outliers.

Figure 12 shows the effect on the number of individuals in the final repertoire when disabling a given input to the controller. The average number of individuals in the repertoire with one input disabled is compared to the repertoire utilizing all the information available. Disabling the nearest neighbor, least frequently visited neighboring square, or the average predicted location results in significant reduction in number of individuals in the repertoire. This is tested using a Rank-Sum statistical test, comparing against repertoires evolved using the full set of inputs.

## 5. Discussion

A key motivation in this work was to enable the top-down definition of swarm behaviors and to develop a framework for automating behavior generation. Operators, or even researchers, designing and using swarms are commonly interested in the macroscopic behavior of the swarm, not the low-level interaction between swarm agents. How to find the low-level controllers that enable a given high-level behavior is an unresolved question in swarm research. To this end, this work contributes another method of generating behaviors based on high-level goals or metrics. The methods presented here are powerful, but not complete. It is easy to develop behaviors that are fluid or organic. However, this framework would be less suitable for producing behaviors that require agents to assemble into pre-defined patterns. This is a trade-off, as the controllers in this work were made to be simplistic by design. Constraints on single agent motion and movements are soft, making rigid formations hard to achieve. To enable more complex behaviors may require controllers with an internal state machine, or at the very least, more complex rule-based structures. This would further add to the time required to optimize or evolve the controllers.

A multi-function swarm is a swarm in which all agents of a swarm might contribute to several applications or functions at the same time. While some applications may require specialized hardware, making a heterogeneous swarm more suitable to the task, multi-function swarms seek to tackle tasks that are continuous in nature, where a single type of swarm agent in sufficient numbers is enough to tackle the task. The challenge for a multi-function swarm is to incorporate the many, potentially conflicting, requirements from each application. Each application or function contributes some constraint or requirement to the swarm behavior for optimal performance. As more constraints or applications are added, it might seem intuitive that the resulting set of viable solutions are reduced. Going from two applications (exploration and networking) to three applications (exploration, networking and geolocation) this did not seem to be the case—rather the opposite. In a previous work a single repertoire could hold 608 solutions (Engebråten et al., 2018b), compared to the average of 2,031 solutions per repertoire in this work. The combination of an open-ended evolutionary method and a multi-function behavior repertoire seems to enable the evolution of repertoires that truly span the entire range of viable solutions. This is primarily due to MAP-elites, which does not seek solutions at the extreme of each objective like a traditional multi-objective optimization. Instead, by attempting to illuminate the search landscape it is possible to show all the different trade-offs between applications and how performance in different applications interact. This avoids the pitfall of an empty set being the only viable result when constraints from each application are applied, and highlights the potential of a multi-function swarm system.

Re-evaluating a whole repertoire highlights the issue of combining stochastic metrics with MAP-elites or in general, Quality-Diversity methods. In these experiments, re-evaluation of the entire repertoire resulted in a reduction in repertoire size of up to 76.2%. Through the use of 5-evaluations per individual this was reduced to 63.0%, but this is still a drastic reduction in the size of the original repertoire. Multiple evaluations contribute to tackling this challenge. However, as seen from Figure 11, there is still room for the behaviors to seemingly move within the repertoire. By doing these experiments it is possible to highlight that noise in Quality-Diversity methods is a challenge. Noise must be considered when designing experiments to discover the true shape of the underlying repertoire. In traditional genetic algorithms it is common to operate with a limited number of elites. In terms of MAP-elites all solutions in the repertoire become an elite and none are re-evaluated. One idea could be to enforce a shelf-life on solutions, i.e., throwing out solutions that are too old as determined by when their last evaluation occurred. Another option would be to require re-evaluation if the solution has persisted in the repertoire for too long. This might remove solutions that get a lucky draw from the a single or a few simulations runs. More research is required to figure out the appropriate measure in order to fully address this issue.

Behavior characteristics can be challenging to design, specifically because evolutionary methods excel at finding ways to exploit metrics without actually providing the intended, or desired, type of behaviors. In this work, exploration is measured by the median visitation count. This was a result of previous experiments using an average metric, which resulted in behaviors merely alternating between two cells instead of actually exploring the area. This provided the same gain in metrics, but did not actually allow for the type of behaviors that were desired. For geolocation, the metric used is the variance of the predicted location. The assumption is that variance decreases as the estimated mean converges on the true mean. This is often the case but not always. In some very specific cases it is possible to introduce a skew or a bias, where there is a fairly low variance in predictions while most of the predictions are in the wrong place (Engebråten, 2015).

## 6. Conclusion

This paper presents a concept for automated behavior generation using evolution for a multi-function swarm. Multi-function swarms have the potential to allow for a new type of multi-tasking previously not seen in swarms. With complex environments and scenarios, it is likely that the operator's needs and requirements will change over time, and as such, the swarm should be capable of adapting to these changes. The viability of evolving large repertoires of behaviors is demonstrated using MAP-elites. These behaviors can be considered behavior primitives that allow for easy adaptation of the swarm to new requirements. It can potentially even be achieved on the fly if simple messages can be broadcast to the entire swarm. This allows the operator to change the behavior based on a change in preferences, desires or other external events.

Noise is a challenge in MAP-elites. The combination of a greedy algorithm and noisy metrics can result in repertoires that do not reflect the true shape and properties of the underlying system. In this work multiple evaluations is used to reduce the effect of noise. Noise in metrics may enable poorly performing solutions with high variance to outperform better solutions with lower variance due to a lucky draw. Multiple evaluations is not a complete solution, however this study highlights that noise must be considered when applying Quality-Diversity methods, such as MAP-elites.

It is possible to investigate the effect each input to a controller has on the swarm performance through parameter ablation. The three most important inputs for this type of artificial physics controller was the nearest neighbor, the least frequently visited neighboring cell and the average predicted emitter location. Results indicate that more information might be better, but more research is required to conclude with certainty.

Similarly, to the adaptation mechanism presented by Cully et al. (2015), it is possible to use a repertoire of behaviors as a way of rapidly adapting to hardware faults or communication errors. In real-world systems communication is unreliable. Having a repertoire could in the future enable even more graceful degradation of performance than what is currently innate within swarms. Optimizing repertoires not only for the three application (exploration, network coverage, and localization), but also for varying degrees of allowed communication could bolster the resilience of swarm system. This is future work.

## Data Availability Statement

The datasets generated for this study are available on request to the corresponding author.

## Author Contributions

SE designed and implemented the framework, performed the analysis, and wrote most of the text. JM and OY provided constructive high level feedback, insights and perspectives on the work, and early drafts of the paper. KG contributed feedback and suggestion during the concept development stage, written changes, and improvements to the paper. All authors contributed to the article and approved the submitted version.

## Conflict of Interest

The authors declare that the research was conducted in the absence of any commercial or financial relationships that could be construed as a potential conflict of interest.

## Footnotes

^1^https://github.com/ForsvaretsForskningsinstitutt/Paper-framework-for-multi-function-swarms
